# Systemic Immune Dyshomeostasis Model and Pathways in Alzheimer’s Disease

**DOI:** 10.3389/fnagi.2019.00290

**Published:** 2019-10-23

**Authors:** Puneet Talwar, Suman Kushwaha, Renu Gupta, Rachna Agarwal

**Affiliations:** ^1^Department of Neurology, Institute of Human Behaviour and Allied Sciences (IHBAS), University of Delhi, Delhi, India; ^2^Department of Microbiology, Institute of Human Behaviour and Allied Sciences (IHBAS), University of Delhi, Delhi, India; ^3^Department of Neurochemistry, Institute of Human Behaviour and Allied Sciences (IHBAS), University of Delhi, Delhi, India

**Keywords:** aging, inflammation, autoimmune, amyloid, tau, prevention

## Abstract

Alzheimer’s disease (AD) still remains an enigma for researchers and clinicians. The onset of AD is insidious, gradually progressive and multifactorial. The recent accumulated scientific evidences suggests that the pathological changes resemble the autoimmune-driven self-sustaining inflammatory process as a result of prolonged oxidative stress and immune dyshomeostasis. Apart from aging, during life span various other factors—mainly environmental, lifestyle, chronic stress, polymicrobial infections and neuroendocrine functions—affect the immune system. Here, we provide crosstalk among “trigger insults/inflammatory stimulus” i.e., polymicrobial infection, chronic stress, pro-inflammatory diet and cholinergic signaling to put forward a “Systemic Immune Dyshomeostasis” model as to connect the events leading to AD development and progression. Our model implicates altered cholinergic signaling and suggests pathological stages with various modifiable risk factors and triggers at different chronological age and stage of cognitive decline. The search of specific autoantibodies for AD which may serve as the suitable blood/CSF biomarkers should be actively pursued for the early diagnosis of AD. The preventive and therapeutic strategies should be directed towards maintaining the normal functioning of the immune system throughout the life span and specific modulation of the immune responses in the brain depending on the stage of changes in brain.

## Introduction

Alzheimer’s disease (AD), the most common dementia subtype, affected 50 million individuals globally in 2018. It is a major cause of disability, poor quality of life and care giver’s burden (Patterson, 2018). As the world population is aging, the numbers of patients with AD are expected to increase in the next decade worldwide. The socio-economic burden of AD will pose a tremendous health risk at the global level in the coming decades. Despite several decades of research, the precise pathophysiological process underlying AD is still unelucidated. To date, there is a lack of definitive biomarkers for early prediction as well as absence of disease-modifying therapy.

Although the role of amyloid beta (Aβ) and hyperphosphorylated tau has explained the molecular pathogenesis cascade in AD, the quest for a disease-modifying drug and biomarker(s) for early diagnosis is still on. The search for the causative components of AD has been a matter of debate among researchers in the quest for optimal therapeutic and preventive strategies. There is evidence available demonstrating an association of AD with educational level, genetics and vascular, cultural and psychosocial factors from different epidemiological, neuroimaging and neuropathological studies establishing the multifactorial nature of this complex debilitating disorder (Talwar et al., 2016; Scheltens et al., 2016).

Age can be considered as the most reliable risk factor for AD. With aging, the immune system undergoes a cascade of remodeling and restructuring called immunosenescence, leading to an increase in autoimmunity. Various other factors such as environmental, genetic, neuroendocrine and medical factors along with the contribution of sociodemographic and cultural factors also play an important role in immune alteration (Najjar et al., 2013). The degeneration of the cholinergic system has been implicated in the etiology of AD, dementia and aging. The cholinergic hypothesis, first put forward in 1982, was modified after newer data emerged from translational and clinical research highlighting the failure of the cholinergic system in AD pathogenesis (Bartus et al., 1982; Hampel et al., 2019).

**Figure 1:**
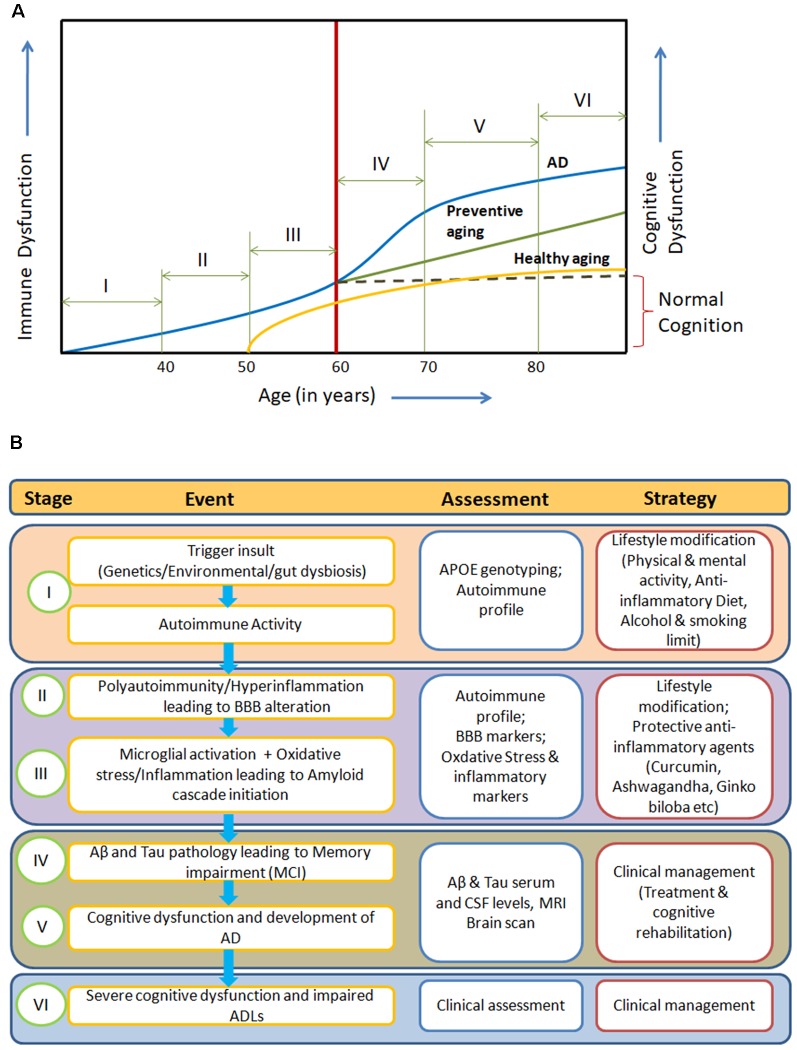
**(A)** Systemic Immune Dyshomeostasis Model representing stages leading to the development and progression of Alzheimer’s disease (AD). **(B)** Possible assessment and prevention strategy at different stages of AD model. The blue line in **(A)** represents stages and events leading to AD. In response to different trigger insults, over years, the autoimmune activity begins to increase in the humans due to immunosenescence gradually from around 30 years of age (Stage I). Further, progression in immune impairment due to repeated trigger insults may be attributed to polyauoimmunity/hyperinflammation, which leads to blood-brain barrier (BBB) dysfunction and then amyloid cascade activation through microglial activation and oxidative stress (stage II and III). Inflammation, autoimmune activity and BBB alterations (at stages I and II) are known to be associated with amyloid oligomers and tau pretangles. Abeta and tau pathology in the form of soluble oligomers and pretangles or paired helical fragments could be present at middle age (40–50) without causing clinical cognitive decline (stages II and III). In the absence of any preventive action, irreversible immune dysfunction cascade begins along with amyloid and tau pathological changes (around 60 years), leading to cognitive impairment (Stage IV). Activation of dyshomeostatic events leads to faster decline in immune functions and cognitive functions, resulting in the development of AD (Stage V). These systemic immune dyshomeostatic events can be controlled through periodic assessment and lifestyle modifications along with the use of neuroprotective/immune strengthening anti-inflammatory agents, which signifies “Preventive aging” (green line). The “Healthy aging” (yellow line) shows the ideal condition where there is neither any predisposing risk factor nor exposure to pathological trigger insults. Only aging-related immune dysfunction appears at late age with slow decline and mild cognitive impairment in cognitive functions.

The manifestation of clinical symptoms ~20 years after initiation of the pathological process, the degeneration of specific neuronal subtypes (cholinergic neurons) with the presence of inflammation and oxidative stress points towards a gradual systemic homeostatic dysfunction in AD. Here, we propose an integrated model for AD pathogenesis to understand its development, progression and prevention (Figures 1A,B).

## Aα–Tau–ApoE: Direct or Indirect Players

In the last several years, numerous AD therapeutic molecules have been tested in hundreds of clinical trials (Hung and Fu, 2017). Most were focused on Aβ as a target based on the most widely accepted amyloid hypothesis. However, expected outcomes were not observed due to various reasons such as poor efficacy, occurrence of adverse events, late AD stage patients, and poor brain permeability (Cummings, 2018; Cummings et al., 2018). This led to a debate on the role of Aβ as a central player in the AD pathogenesis (Tse and Herrup, 2017; Morris et al., 2018). The presence of Aβ in the brain of cognitively normal healthy individuals also contradicts the potential of Aβ to cause AD alone (Neuropathology Group, 2001). Further, it is believed that AD develops gradually and Aβ accumulation starts 10–20 years before the onset of clinical AD symptoms. The role of tau protein has always been shown in association with Aβ as tau alone cannot lead to cognitive decline resulting in AD phenotype (King et al., 2006; Talwar et al., 2016; Bennett et al., 2017). The mechanism of amyloid metabolic pathways such as clearance is still emerging. Interestingly, novel mechanisms of amyloid clearance have emerged. de Leon et al. (2017) recently showed that the human nasal turbinate (NT) is a part of the CSF clearance system. CSF clearance abnormalities in lateral ventricle and superior NT are found in AD, and decreased ventricular CSF clearance is associated with increased brain Aβ accumulation (de Leon et al., 2017). Pappolla et al. (2014) demonstrated lymphatic Aβ clearance *in vivo* using Alzheimer’s transgenic mice. The authors also showed that Aβ levels in lymph nodes increase over time, mirroring the increase of Aβ levels observed in the brain (Pappolla et al., 2014). Additionally, the amyloid deposition follows an extremely complicated aggregation process, and key aspects of amyloid-β oligomer are still unelucidated (Brannstrom et al., 2018; Cline et al., 2018). Further, there is also a presence of Aβ oligomer heterogeneity and fibril polymorphism (Xue et al., 2019).

The Apolipoprotein E (*APOE*ε4) allele appears to be a risk factor accounting for only 10%–25% of AD cases and not an invariant cause of AD, as more than half of AD cases do not have the high-risk E4 allele, indicating that other environmental or genetic factors may need to be concurrently acting with this allele to cause AD (Hyman et al., 1996; Lambert et al., 2013; Haines, 2018; Jiang et al., 2019). APOE isoforms have been shown to differentially modulate the Aβ-dependent and independent pathways (Yamazaki et al., 2019). However, the pathogenesis involved in sporadic AD without *APOE*ε4 and through Aβ-independent pathways remains elusive. In an interesting case report, Mak et al. (2014) reported a patient lacking functional ApoE protein with normal vision, retinal cognitive and neurological functions, with no abnormal findings on brain magnetic resonance imaging (MRI) and with normal CSF levels of Aβ and tau proteins (Lane-Donovan and Herz, 2014).

## Systemic Immune Dyshomeostasis Model: An Integrated Model for AD Pathogenesis

We envisage AD as a Systemic Immune Dyshomeostasis disorder that manifests after encountering a “trigger insult” which can be either through internal (genetic predisposition, neuroendocrine and gut dysbiosis) or external (stress, infections, diet, lifestyle, drugs, metal toxicity, alcohol and pollution) factors leading to blood-brain barrier (BBB) dysfunction (Dosunmu et al., 2007; Xu et al., 2015; Talwar et al., 2019).

In the beginning, the trigger insults are primarily responsible for the initiation of autoimmune activity or auto-inflammatory process (Temajo and Howard, 2014). Further, repeated challenges by these triggers lead to polyautoimmunity or hyperinflammation (Anaya, 2014; Gul, 2018). These systemic alterations along with ageing and immunosenescence result in BBB dysfunction, allowing serum proteins to reach CNS or formation of immune passage allowing peripheral T lymphocytes to cross the BBB (Carson et al., 2006; Sonar and Lal, 2018), leading to microglial activation which in turn initiates neuronal oxidative stress and inflammatory process, activating amyloid cascade in cyclic mode causing progressive neurodegeneration. The mild cognitive impairment occurs once the β-amyloid monomers convert into soluble oligomers and hyperphosphorylation of tau protein occurs, which gradually proceeds to AD with the deposition of amyloid plaques and formation of neurofibrillary tangles (Jeong, 2017). In advanced stages of AD, the pathology covers the entire neocortex with complete degeneration of acetyl cholinergic neurons, cortical atrophy and enlargement of ventricles, which results in marked phenotypic, personality and behavior changes along with cognitive dysfunction and impaired activities of daily living (ADLs; Figure 1A).

Several studies have reported a higher risk of AD with bacterial and viral infections (Sochocka et al., 2017; Ashraf et al., 2019; Talwar et al., 2019). The recent antimicrobial protection hypothesis has suggested that polymicrobial infection may be involved in activating the innate immune response leading to Aβ deposition (Gosztyla et al., 2018; Moir et al., 2018). Several studies have proposed potential molecular mechanisms of enhanced Aβ production and reduced Aβ clearance in the context of viral infections, mainly HIV (Pulliam, 2009; Lan et al., 2011). Further, Civitelli et al. (2015) showed that Herpes simplex virus type 1 (HSV1) infection in primary cultures of cortical neurons leads to production and nuclear localization of APP intracellular domain (AICD) *in vitro* (Civitelli et al., 2015). Some emerging literature has pointed towards the presence of different Aβ species in response to various microbial infections (Zhao et al., 2015; Spitzer et al., 2016). Several pathogenic Aβ and Tau isoforms have been reported which may render the drugs targeting these proteins ineffective (Lacovich et al., 2017; Dujardin et al., 2018; Goedert, 2018; Hodgson, 2019).

ApoE has also been implicated in various pathologies, including infection, dyslipidemia, vascular pathologies, apart from AD due to structural isoforms (Urosevic and Martins, 2008; Tudorache et al., 2017). The role of ApoE4 derived antimicrobial peptide analogs has also been reported (Kelly et al., 2007).

Kamer et al. (2009) showed that TNFα and antibodies to periodontal bacteria discriminate between AD patients and normal subjects, indicating a role of bacterial infection and inflammation in AD (Kamer et al., 2009).

In people with chronic stress, such as medical professionals and those in the armed forces, higher risk of AD has been observed (Wang et al., 2012; Veitch et al., 2013; Greenberg et al., 2014; Ridout et al., 2019; Yaffe et al., 2019). Strong evidence is emerging that shows sleep disturbances and/or disorders as an important risk factor in the early pathogenesis of AD (Bubu et al., 2017; Van Egroo et al., 2019). Sleep abnormalities lead to systematic inflammation and an increase in reactive oxygen species (ROS) production, neuronal activity, interstitial fluid (ISF) and CSF tau, and decrease in glymphatic clearance of Aβ protein. Prolonged oxidative stress can initiate or augment the neuropathological process in AD (Ning and Jorfi, 2019). Xie et al. (2013) provided evidence that sleep drives clearance of potentially neurotoxic metabolites, including amyloid, from the adult brain (Malkki, 2013; Xie et al., 2013).

Although there is insufficient evidence associating certain diets with risk of AD, several studies reported higher risk of AD in people consuming western diets rich in meat, sugar and processed foods. A diet rich in plant foods, whole grains, fresh dairy products and fish as included in several dietary patterns such as the DASH, Mediterranean, and Japanese diets has been shown to be protective against AD (Hu et al., 2013; Grant, 2016).

**Figure 2:**
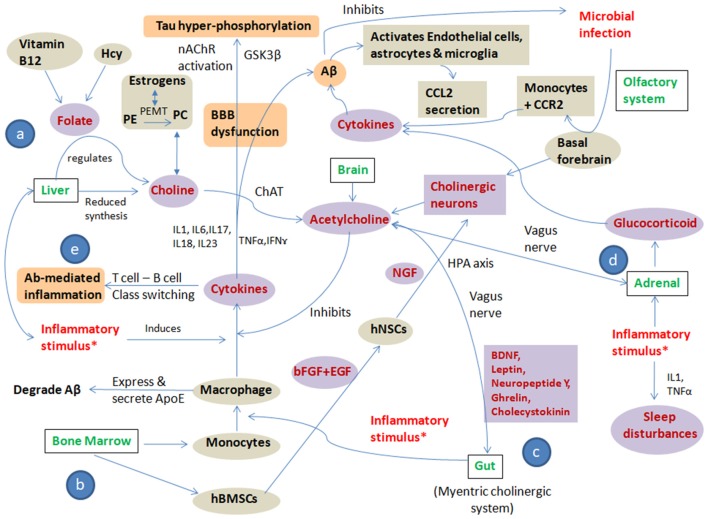
Pathways showing systemic immune dyshomeostasis and the effect of inflammatory trigger insults on cholinergic signaling in AD: the figure shows crosstalk among “trigger insults/inflammatory stimulus” i.e., polymicrobial infection, chronic stress, pro-inflammatory diet and cholinergic signaling to put forward the “Systemic Immune Dyshomeostasis” model as to connect the events leading to AD development and progression. All three trigger insults lead to altered cholinergic signaling pathway in brain, bone marrow, liver and gut by modulating: (a) one carbon (1C) metabolism; (b) bone marrow-derived monocytes and bone marrow-derived mesenchymal stem cells (BMSCs); (c) gut microbiome, hypothalamic pituitary axis; and (d) glucocorticoid regulation culminating in immune alteration and autoimmunity (e). The Violet shaded circles represent the “direct players,” the gray shaded circles represent the “indirect players” and the orange shaded circles represent the “pathological players” in AD pathogenesis cascade. *Infection, stress and inflammatory diet; Hcy, Homocysteine; PE, Phosphatidylethanolamine; PC, Phosphatidylcholine; PEMT, Phosphatidylethanolamine N-methyltransferase; ChAT, Choline O-Acetyltransferase; nAChR, Nicotinic acetylcholine receptors; GSK3β, Glycogen synthase kinase 3 beta; IL, interleukins; TNFα, Tumor necrosis factor alpha; IFNγ, interferon γ; hBMSCs, human bone marrow-derived mesenchymal stem cells; hNSCs, human neural stem cells; bFGF, basic fibroblast growth factor; EGF, Epidermal growth factor; NGF, Nerve growth factor; ApoE, Apolipoprotein E; Aβ, Amyloid beta; CCL2, C-C Motif Chemokine Ligand 2; CCR2, C-C chemokine receptor type 2.

These substantial evidences allow us to suggest that polymicrobial infection, chronic stress and a pro-inflammatory diet are the main “trigger insults/inflammatory stimulus” that cause altered cholinergic signaling leading to systemic immune dyshomeostasis, resulting in AD development and progression. All three trigger insults lead to an altered cholinergic signaling pathway in brain, bone marrow, liver and gut by modulating one carbon (1C) metabolism, bone marrow-derived monocytes and bone marrow-derived mesenchymal stem cells (BMSCs), gut microbiome, hypothalamic pituitary axis and glucocorticoid regulation culminating in immune alteration and autoimmunity. The pathways showing systemic immune dyshomeostasis and the effect of inflammatory trigger insults on cholinergic signaling in AD are represented in Figure 2.

## Crosstalk Between Cholinergic Signaling, Trigger Insults and One Carbon (1C) Metabolism

Adequate levels of acetylcholine (Ach) are essential for normal immune and cognitive functions like alertness, attention, learning and memory. As peripheral Ach cannot cross the BBB, it is primarily synthesized in the basal nucleus of Meynert and medial septal nucleus of basal forebrain from choline and acetyl coenzyme A (acetyl-CoA) for various physiological functions. Choline is also essential for the development and functioning of the hippocampus and frontal cortex, which are mainly responsible for memory and high-level thinking respectively. The choline levels are controlled by one-carbon (1C) metabolism involving folate, DHA, EPA, vitamin B12 and homocysteine in liver (Blusztajn et al., 2017).

Reduced transport of acetyl-CoA into the ER lumen has been shown to cause neurodegeneration along with increased predisposition to inflammation and infections. NAA produced from Acetyl-CoA and aspartate serves as an extensive reservoir of acetate for Acetyl-CoA synthesis, and its loss impairs Acetyl-CoA signaling, leading to neuronal injury (Moffett et al., 2007, 2013). The inhibition of enzyme acetyl-CoA carboxylase (ACC), involved in the conversion of acetyl-CoA to malonyl-CoA, is considered as a potential therapeutic target for different complex disorders including viral infections (Greseth and Traktman, 2014; Merino-Ramos et al., 2015). Previous studies have suggested a role for acetylcholine (Ach) and catecholamines (CAs) in the crosstalk between pathogens and the immune system. The presence of pathogenic signatures modulates cholinergic and catecholaminergic signaling pathways leading to the production of pro-inflammatory cytokines (Weinstein et al., 2015).

Altered synthesis and import of fatty acids—mainly cholesterol, glycerophospholipids and sphingolipids—have been implicated in AD, cancers, obesity, diabetes and viral infections (Merino-Ramos et al., 2015). In the liver, phosphatidylethanolamine N-methyltransferase (PEMT), a transferase enzyme, converts phosphatidylethanolamine (PE) to phosphatidylcholine (PC), which serves as a precursor of choline for Ach synthesis. The PEMT gene is shown to be regulated by estrogen—which increases the PEMT activity—and lower level of estrogen has been implicated in post-menopausal women as a risk factor for AD (Resseguie et al., 2007). A correlation of plasma choline and betaine [choline oxidation product involved in dimethylglycine formation through methylation of homocysteine (Hcy) to methionine (Met)] with serum folate, plasma S-adenosyl-methionine and S-adenosyl-homocysteine has been well established (Imbard et al., 2013). Folate and choline are metabolically interrelated, and their deficiency is associated with increased plasma Hcy concentration as reported in patients with AD (Jacob et al., 1999; da Costa et al., 2005). Diets rich in red and processed meat, fried food, peas and legumes with a lower proportion of whole grains has been shown to alter 1C metabolism, resulting in release of proinflammatory cytokines and accelerated cognitive dysfunction at older ages as also demonstrated by the Whitehall II cohort study (Ozawa et al., 2017).

## Crosstalk Between Cholinergic Signaling, Trigger Insults and Bone Marrow-Derived Monocytes/Bone Marrow-Derived Mesenchymal Stem Cells (BMSCs)

It is well known that most of the proinflammatory cytokines released systemically during sustained inflammatory responses are produced from tissue macrophages and not by circulating monocytes. Higher concentration of pro-inflammatory cytokines modulates synaptic plasticity and neurotransmitter signaling. The crosstalk between the innate immune system and the cholinergic nervous system through synapse requires the α7 subunit of the acetylcholine receptor (AChR). The interaction of cholinergic agonists including nicotine and acetylcholine with the AChR inhibits the production of pro-inflammatory cytokines released by activated macrophages, but not the anti-inflammatory cytokines. The pro-inflammatory cytokine-inhibiting effects of Ach work on monocytes, which express little or no α7 AChR, only at supra-physiological concentrations of cholinergic agonists. This highlights the anti-inflammatory effect of the parasympathetic nervous system (PNS) *via* acetylcholine (Czura et al., 2007).

Trigger insults induce macrophages from bone marrow derived monocytes to secrete proinflammatory cytokines mainly IL-1, TNFα, IFNγ, IL-17, IL-18 and IL-23, which can cross the BBB and induce Aβ generation along with Tau hyperphosphorylation *via* GSK-3β or nicotinic acetylcholine receptors (nAChR) activation. These proinflammatory cytokines have been shown to increase the mRNA expression and synthesis of APP in endothelial and neuronal cells (Chen et al., 2014; Tahmasebinia and Pourgholaminejad, 2017; Alasmari et al., 2018). Higher serum levels of these pro-inflammatory cytokines have been reported in patients with AD (Lai et al., 2017). However, IL-17A, when overexpressed in an AD mouse model, led to increased ABCA1 expression which in turn resulted in reduction of Aβ levels in the hippocampus and cerebrospinal fluid levels (CSF), which may point towards their protective role at initial stages (Yang et al., 2017). Sustained inflammatory stimulus results in hyperinflammation, which results in continuous production of inflammatory cytokines and decreases production of anti-inflammatory cytokines (e.g., IL-4, IL-10, IL-11, IL-13), causing accumulation and deposition of Aβ. This activates a cyclic cascade wherein Aβ induces neuronal damage which activates astrocytes and microglia to produce chemokine C-C Motif Chemokine Ligand 2 (CCL2), resulting in recruitment of more immune effector cells to the site of Aβ deposition. Macrophages differentiated from blood-derived monocytes, phagocytose and clear Aβ plaques more effectively than brain resident microglia (Hohsfield and Humpel, 2015).

In an interesting *in vitro* study, it has been reported that human neural stem cells (hNSCs) from human bone marrow-derived mesenchymal stem cells (hBMSCs) could be differentiated into cholinergic neurons (CNs) using basic fibroblast growth factor (bFGF), epidermal growth factor (EGF) and B27 (media supplement) followed by replacement with nerve growth factor (NGF), suggesting a role of these factors in the generation of cholinergic neurons (Adib et al., 2015). The role of EGF, EGFR and NGF has been previously implicated in AD (Talwar et al., 2014, 2017; Cuello et al., 2019; Fahnestock and Shekari, 2019).

## Crosstalk Between Cholinergic Signaling, Trigger Factors and Gut Microbiome

AD development has also been attributed to gut–brain axis dyshomeostasis and pathogen-derived amyloidogenesis. Chronic inflammatory stimulus in the gut may induce release of proinflammatory cytokines. The endogenous gut microbiome may release amyloid-associated factors such as amyloids, lipopolysaccharide, and serum amyloid A, which may escape from the gastrointestinal (GI) tract and further increase the proinflammatory cytokine levels. Increased BBB permeability due to aging or dysfunction allows the proinflammatory cytokines to enter brain inducing glia reactivity, TLR2/1, CD14 signaling and iNOS increase. Additionally, altered expression of BBB-associated receptor for advanced glycation end products (RAGE), low-density lipoprotein receptor related protein 1 (LRP) receptors and tight junctions facilitate transit of amyloid proteins and leukocytes, leading to activation of NF-κB signaling and an increase of ROS levels resulting in neuroinflammation. Perturbations of gut homeostasis due to trigger insults can also lead to altered GABA, NMDA, and BDNF signaling, causing impaired glucose metabolism and reduced insulin sensitivity, culminating in neurodegeneration and cognitive impairment (Pistollato et al., 2016; Bostanciklioğlu, 2019; Kowalski and Mulak, 2019; Mohajeri, 2019).

The microbiota can modulate events in the periphery and brain by different ways, including cytokine production, vagus nerve activation, neuropeptide and neurotransmitter release, short-chain fatty acids (SCFA), α-Amino-β-methylaminopropionic Acid (BMAA) and lipocalin-2 release. These signals reach the brain and influence the microglial maturation and activation. Activated microglia facilitate immune surveillance, regulate hypothalamic-pituitary-adrenal (HPA) axis, and manages synaptic pruning and clearance of debris (Rea et al., 2016). In a recent interesting study by Werbner et al. (2019), chronic social stress is shown to promote the expression of virulent genes in the murine gut microbiota which activates the immune response, compromising tolerance to self and resulting in increased risk for autoimmune disorders in susceptible individuals (Werbner et al., 2019).

## Crosstalk Between Cholinergic Signaling, Trigger Factors and Glucocorticoid Hormones

Inflammatory stimulus may induce the release of glucocorticoid hormones as a consequence of HPA axis activation, which can, in turn, activate the brain microglia as well as influence release of proinflammatory cytokines and monocyte trafficking from the periphery to the CNS (Rea et al., 2016). Glucocorticoids (mainly cortisol) primarily target the cortical and limbic brain regions, including the hippocampus, which contain the basal forebrain cholinergic neuronal projections and are involved in the etiology of stress, cognitive aging and neurodegenerative diseases including AD.

Trigger insults (stress) affect the release of ACh, glucocorticoids and their receptor affecting cognitive processes and functions. During stress, interaction between glucocorticoids and the cholinergic system contributes to degeneration of basal forebrain cholinergic neurons, leading to progressive cognitive decline with aging and in AD (Paul et al., 2015). Physiological stress has been shown to alter the post-translational modification of acetylcholinesterase (AChE) by shifting it from healthy (AChE-S splice variant) to a less stable AChE-R variant. Stress, inflammation and iron have been reported to modulate the microglial phenotype. Additionally, evidence showing crosstalk between α7nAChR and the ferroportin signaling pathway is also present (Cortes et al., 2017).

Basal forebrain cholinergic neurons also mediate HPA axis activation and regulate rapid eye movement (REM) sleep. The HPA axis can be activated directly by the cholinergic agonists and indirectly *via* an inflammatory stimulus including chronic stress (Krieg and Berger, 1989). In 1995, Korth proposed the theory of co-evolution between sleep and BBB, which stated that “sleep primarily evolved to protect the brain against a wakefulness-dependent increase in the permeability of the BBB.” It implies that sleep is regulated by bacterial cell wall constituents from gut microbiota through BBB permeability and cytokine production (Korth, 1995).

## Autoimmunity in AD Pathogenesis

The presence of antigenic homology between Aβ42 with pathogens (including viruses), food antigens such as microbial transglutaminase (mTG), β-NGF, BDNF, component BBB antigens and enteric neuronal antigens has been reported. These Aβ42 cross-reactive autoantibodies (aAb) have been shown to cause neuronal degeneration in individuals with compromised BBB (Vojdani and Vojdani, 2018). There are numerous aAb detected in human sera, and some of them have specific roles in the pathogenesis of AD. A comprehensive list of aAb associated with AD is provided in Supplementary Table S1. These antibodies are directed against Aβ and tau proteins, neurotransmitters, glial markers, lipids and cellular enzymes. The natural antibodies are produced by human beings against Aβ and tau proteins, which was the basis of immunotherapy. The patients with autoimmune disorders such as rheumatoid arthritis (RA) have been shown to have higher risk of developing cognitive dysfunction leading to AD (Li et al., 2018). Several studies have demonstrated the involvement of autoimmune factors such as Aβ aAb, anti-angiotensin 2 type 1 receptors (anti-AT1R) aAb, intermediate neurofilament heavy (NFH) protein as primary self-antigen (pSAg), immunoglobin (Ig) positive neurons and NGF reduction in AD pathogenesis (Supplementary Table S1). In the study by Nagele et al. (2011), the authors identified a panel of AD autoantibody markers with a sensitivity of 96.6% and a specificity of 92.5%. In a study by Tuszynski et al. (2015) involving 10 AD subjects and NGF gene therapy, neuronal regeneration, tissue growth and improvement in cognitive functions were reported. The proNGF converts into mature NGF in the brain, which stops in AD, leading to Aβ formation and brain inflammation resulting in neuronal degeneration and dysfunction. Further, the role of NGF in selectively protecting acetylcholine-activated neurons has been demonstrated, indicating potential for its use in the treatment of AD (Aloe et al., 1994).

As many aAb have been detected in AD patients contributing to the pathogenesis of AD and also displaying the protective role, these antibodies can serve as corroborative diagnostic or prognostic biomarkers. The presence of neuronal aAb in combination with BBB disruption is the main contributing factor in AD pathogenesis. The identification of pSAgs is a challenge, as the autoimmune process continues for years before manifesting as disease. In recent years, hippocampal self-antigens have been found in the sera of patients with autoimmune encephalitis (Graus et al., 2016). The identification of pSAgs depends on the humoral immune system. The optimum function of normal homeostasis, maintenance and proper neuronal function is modulated by intact BBB. Disruption in the BBB allows serum proteins or antigens to reach the brain regions. Antibodies to different brain proteins have been identified which are produced after an autoimmune response. These specific antibodies act as mediator to cause inflammation, resulting in severe neuronal and glial dysfunction. In neurodegenerative disorders, the chronic inflammation is associated with specific brain areas. The T lymphocyte mediated degeneration of cholinergic myelinated large axons in the fore brain leads to severe cognitive deficits (Lueg et al., 2015). The crosstalk between activated B and T lymphocytes regulates the pathogenic antibody responses through the antibody class-switching and glycosylation pattern (Ludwig et al., 2017).

## The Way Forward: Preventive Strategies and Multitherapy Approach

There are only four FDA-approved drugs for AD—namely Donepezil, Rivastigmine, Galantamine, and Memantine. All these medications only treat the symptoms of AD and neither stop nor reverse the cognitive decline. At present, there is no disease-modifying treatment available for patients with AD (Vojdani and Vojdani, 2018). Although it is presently not possible to reverse the cognitive decline in the clinically diagnosed patients with AD due to neuronal degeneration and atrophy in brain regions, it is possible to increase the resilience of the immune system in advance to counter or delay the effect of “trigger insult.”

The possible way to lower the future increase in burden of AD is to focus on the prevention strategy. As AD is considered a multi-factorial disorder, the strategy needs to be multi-focal, with a central focus on immune strengthening process targeting population of age less than 40 years (primary prevention). The preventive measures include regular physical activity, meditation, anti-inflammatory diet and use of supplements with potential antioxidant, neuro-protective and immune-strengthening properties.

Lifestyle changes—primarily physical activity (exercise, yoga), sleep hygiene, meditation, social engagement and educational status—also play an important role in maintaining cognitive functions and cognitive reserve over the years (Shatenstein et al., 2015; Xu et al., 2015). An anti-inflammatory diet which includes fresh fruits such as berries, spices (garlic, ginger, turmeric, black pepper), gluten-free products, fermented food items, fish and red wine is believed to promote healthy ageing (Daulatzai, 2015). The periodic use of supplements—mainly omega-3 fatty acids [eicosapentaenoic acid (EPA) and docosahexaenoic acid (DHA)], vitamin B12, vitamin E, folic acid, curcumin with black pepper (piperine), grape seed extracts (gallic acid and catechins), Withania Somnifera (withanolide withaferin A), Ginko Biloba (ginkgolide), barberry (berberine), ginseng (Gintonin), Resveratrol and probiotics—can prevent autoimmune activity and inflammation through different mechanisms and can also be beneficial in preventing early onset of cognitive impairment among at-risk individuals (Daulatzai, 2015; Viña and Sanz-Ros, 2018; Wang et al., 2018). These lifestyle modifications may deal with trigger insults and maintain the immune homeostasis, preventing or delaying BBB dysfunction and allowing cross-reactive antibodies to enter into the CNS through cerebral vasculature. The integrated combination therapy approach targeting different stages of the proposed pathological pathway is needed to prevent, delay and/or control the development and progression of AD (Figure 1B).

Recent meta-analysis studies have shown lower acetylcholine, GABA, pyruvate, DHA, choline, vitamin B12, C, E and folate levels in AD (de Wilde et al., 2017; Manyevitch et al., 2018). Supplementation with choline compounds has been associated with improvement in cognitive functions through regulation of epigenomic activities including brain-specific histone modifications and DNA methylations and with alterations in the expression of genes associated with learning and memory processing (Blusztajn et al., 2017). However, a few studies have shown no cognitive improvement despite higher blood levels of choline in recipients (Sanchez et al., 1984; Amenta et al., 2001). The probable reason could be low acetyl-CoA levels as both choline and Acetyl-CoA are essential for the synthesis of acetylcholine and improvement in cognitive functions. Acetate, pyruvate and ketone bodies can also be used as acetyl-CoA precursors (Nakamura et al., 1970; Pietrocola et al., 2015). Frost et al. (2014) have shown that acetate supplementation can activate acetyl-CoA carboxylase, leading to an increase in the levels of acetyl-CoA in the brain (hypothalamus). Administration of pyruvate after severe hypoglycemia has been reported to reduce neuronal death and resulting cognitive impairment (Suh et al., 2005; Zhou et al., 2012). Ketone bodies—mainly water-soluble acetoacetate and beta-hydroxybutyrate molecules—which are produced in the liver and can cross the BBB and reconverted to acetyl-CoA.

To control inflammation, curcumin with piperine as part of the supplementation is essential, as it has been shown to downregulate human TNF-α levels in a systematic review and meta-analysis involving randomized controlled trials (Sahebkar et al., 2016). In addition, other important supplement candidates are Ginkgo Biloba (Increase NGF), Withania Somnifera (steroid-like activity), Glycyrrhiza glabra or Tripterygium wilfordii [heat shock protein 90 (Hsp90) inhibitors], Centella asiatica (increases neurite outgrowth in the presence of NGF), vitamin D with zinc and drug selegiline (elevates NGF, BDNF, and GDNF) (Aloe et al., 1994; Sehgal et al., 2012; Berti et al., 2015; Dal Piaz et al., 2015; Gray et al., 2017; Puttarak et al., 2017; Campanella et al., 2018; Farooqui et al., 2018; Kandiah et al., 2019; Park et al., 2019; Talwar et al., 2019).

## Limitations

The hypothesis involves multiple systems of the body at different points of time, which is difficult to study simultaneously. However, it can be validated through carefully designed longitudinal studies assessing multiple parameters in precisely phenotyped cohorts. Further, immune function is a subject-dependent dynamic entity which varies in response to several internal and external stimuli, and controlling for them in the ageing human population is extremely difficult. Also, complex genetic and epigenetic processes involved in AD may act as limiting factors in the elucidation of underlying neurodegenerative mechanisms in ethnically different phenotypic groups. However, context-dependent underlying physiological mechanisms could be elucidated through *in vivo* and *in vitro* studies. The hypothesis may also hold true for gradually progressive late-onset disorders with multi-physiological system involvement such as Parkinson’s disease and cannot be seen exclusively as AD-specific.

## Conclusion

Several hypotheses have been put forward in the last 100 years, but the precise etiology of AD pathogenesis is still unelucidated. In the present review, we have presented the crosstalk among “trigger insults/inflammatory stimulus,” i.e., polymicrobial infection, chronic stress, pro-inflammatory diet and cholinergic signaling and proposed a model that potentially connects the events leading to AD development and progression. We envisaged systemic immune dyshomeostasis as a multifactorial combination of age- and immune-related changes that modulate the body and brain functions, effectively enabling opportunistic pathobiological alterations leading to AD pathology. The review highlights some of the recent findings on how different inflammatory responses exacerbate neurodegenerative processes associated with AD and how the systemic immune changes, along with a litany of other processes, including chronic stress, cholinergic signaling defects, polymicrobial invasion and other chronic insults, may be responsible for many of these changes. We propose AD as an inflammation-driven self-sustaining autoimmune process and timely immune strengthening interventions may be instrumental in preventing morbidity and mortality. The preventive strategies should be directed towards maintaining the normal functioning of the immune system, and therapeutic strategies should focus on a multitherapy approach.

## Author Contributions

PT and SK conceived and wrote the manuscript. Both authors read and approved the final manuscript. RA and RG were involved in improving, cross-checking and proofreading of the manuscript.

## Conflict of Interest

The authors declare that the research was conducted in the absence of any commercial or financial relationships that could be construed as a potential conflict of interest.
