# Supplementary material for: Systemic Immune Dyshomeostasis Model and Pathways in Alzheimer’s Disease
Source: Front Aging Neurosci. 2019 Oct 23;11:290. doi: 10.3389/fnagi.2019.00290 (PMC6838686; doi:10.3389/fnagi.2019.00290)
Supplement: Supplementary file 1 [file Table_1.DOCX]

**Table S1: List of Autoantibodies implicated in AD or dementia subtype along with their diagnostic potential**

| **S.No.** | **Autoantibody Target** | **Antibody** | **Functional category** | **Association** | **Serum/Plasma/CSF** | **Association of Aab levels** | **Specificity (Diagnostic potential)** |
| --- | --- | --- | --- | --- | --- | --- | --- |
| 1 | Aβ42 [[1](#_ENREF_1)] | ― | Protofibrils | AD | Plasma | Increased Number of Plasma B Cells | **Specific** |
| 2 | Aβ [[2-4](#_ENREF_2)] | IgG | Immune complex | AD, MCI | Serum, CSF, Brain tissue | Increased | **Specific** |
| 4 | Aβ (25-35) [[5](#_ENREF_5)] | IgG | Oligomers | AD (mild-moderate) | Serum | Increased | **Specific** |
| 5 | Cross-linked beta-amyloid protein species (CAPS) [[6](#_ENREF_6)] | IgG | Cross-linked protein | AD | Plasma | Decreased | **Specific** |
| 6 | α1 and β2 adrenergic receptors [[7](#_ENREF_7), [8](#_ENREF_8)] | IgG | Receptor | AD and VaD |  | Increased | Non-specific |
| 7 | MAST1, Enah, MAO-A, X11/MINT1, HGF, SNX14, ARHGAP 11A, APC, and CENTG3 [[9](#_ENREF_9)] | IgG | ― | AD | Serum | Increased | ― |
| 8 | Angiotensin 2 Type 1 Receptor (AT1R) [[10](#_ENREF_10)] | IgG | Receptor | AD | CSF | Increased | **Specific** |
| 9 | Phospholipid (PL) [[11-13](#_ENREF_11)] | IgG | Lipid | AD and MCI | CSF, Serum | Decreased in AD; Increased in MCI | Non-specific (Stroke [[14](#_ENREF_14)], DVT [[15](#_ENREF_15)]) |
| 10 | Galanin and α-MSH [[16](#_ENREF_16)] | IgG | Neuropeptides | AD | CSF | Increased | **Specific** |
| 11 | ATP synthase or Ecto-F1-ATPase (Asabs) [[17](#_ENREF_17), [18](#_ENREF_18)] | IgG | Integral protein | AD | Serum, CSF | Increased | Non-specific (Cancer [[19](#_ENREF_19), [20](#_ENREF_20)]) |
| 12 | α7(1-208) nAChR [[21](#_ENREF_21)] | IgM | Receptor | AD | Plasma | Increased | Non-specific (Rasmussen encephalitis [[22](#_ENREF_22)]) |
| 13 | N-truncated pyroglutamate Aβ (pGluAβ) [[23](#_ENREF_23)] | IgM | Immune complex | AD | Plasma | Decreased | **Specific** |
| 14 | Glutamate [[24-26](#_ENREF_24)] | IgM and/or IgA | Neurotransmitter and Receptors | AD | Plasma | Increased | Non-specific (Encephalopathies [[27](#_ENREF_27)], Multiple sclerosis and Rasmussen's encephalitis [[28](#_ENREF_28)]) |

**Table S1: Continued**

| **S.No.** | **Autoantibody Target** | **Antibody** | **Functional category** | **Association** | **Serum/Plasma/CSF** | **Association of Aab levels** | **Specificity (Diagnostic potential)** |
| --- | --- | --- | --- | --- | --- | --- | --- |
| 15 | S100b [[5](#_ENREF_5), [29](#_ENREF_29), [30](#_ENREF_30)] | IgG | Calcium binding protein | AD (moderate-severe),VaD, SDAT | Serum | Increased | Non-specific (Depressive disorder, epilepsy, multiple sclerosis, and Parkinson's disease [[31](#_ENREF_31)]) |
| 16 | Dopamine (DA)[[5](#_ENREF_5)] | IgG | Neurotransmitter | AD (moderate-severe) | Serum; Plasma | Increased | Non-specific (Autoimmune movement and psychiatric disorders [[32](#_ENREF_32)]) |
| 17 | Serotonin (5-HT)[[33](#_ENREF_33)] | IgG | Neurotransmitter | AD (mild) | Serum; Plasma | Increased | Non-specific (Schizoaffective psychosis, Chronic alcoholism, and Rheumatoid arthritis [[34](#_ENREF_34)]) |
| 18 | Glial fibrillary acidic protein (GFAP) [[29](#_ENREF_29), [35](#_ENREF_35), [36](#_ENREF_36)] | IgG | Intermediate filament (IF) protein | AD and VaD | Serum, CSF | Increased | Non-specific (Autoimmune diabetes [[31](#_ENREF_31), [37](#_ENREF_37)]) |
| 19 | Tubulin [[35](#_ENREF_35)] | IgG | Globular protein | AD | Serum, CSF | Increased | Non-specific (Behcet's Disease [[38](#_ENREF_38)], SLE [[39](#_ENREF_39)], Multiple sclerosis [[40](#_ENREF_40)]) |
| 20 | Histones [[41](#_ENREF_41)] | IgG | Chromosomal proteins | AD, VaD and SDAT | Serum | Increased in VaD and SDAT | Non-specific (SLE [[42](#_ENREF_42)]) |
| 21 | Neuron specific enolase [[30](#_ENREF_30)] | IgG | Enzyme | AD and SDAT | Serum | Increased | Non-specific (Glaucoma[[43](#_ENREF_43)]) |
| 22 | Prolactin cell or Lactotropes [[44](#_ENREF_44)] | IgG | Prolactin producing | AD | Serum | Increased; No change | Inconsistent [[45](#_ENREF_45)], Non-specific (SLE [[46](#_ENREF_46)], RA [[47](#_ENREF_47)]) |
| 23 | Tau [[48](#_ENREF_48)] | IgG | Neuron-specific cytoskeletal proteins | AD | Serum | Decreased | Specific |
| 24 | Tau and Ptau [[49](#_ENREF_49)] | IgG and IgM | Neuron-specific cytoskeletal proteins | AD | Serum | Increased | **Specific** |
| 25 | Intrathecal Tau and Heavy subunits of neurofilaments (NFH) [[50](#_ENREF_50)] | IgG | ― | AD | CSF/serum | Increased | **Specific** |

**Table S1: Continued**

| **S.No.** | **Autoantibody Target** | **Antibody** | **Functional category** | **Association** | **Serum/Plasma/CSF** | **Association of Aab levels** | **Specificity (Diagnostic potential)** |
| --- | --- | --- | --- | --- | --- | --- | --- |
| 26 | Heavy subunits of neurofilaments (NFH) [[48](#_ENREF_48)] | IgG | Neurofilament protein | AD | Serum | Decreased | **Specific** |
| 27 | N ε-(Carboxyethyl) lysine (CEL) [[51](#_ENREF_51)] | IgM | A kind of Advanced glycation end products (AGEs) | Early AD | Serum | Increased | **Specific** |
| 28 | Serotonin receptors [5-HT2AR , 5-HT2CR and 5-HT7R], vascular endothelial growth factor receptor 1 [VEGFR1], immune-receptors (Stabilin-1] and C5aR1 [[52](#_ENREF_52)] | IgG | Receptors | AD | Serum | Increased | **Specific** |
| 29 | Gangliosides (GD1b) [[53](#_ENREF_53)] | IgM | Sialic acid-containing glycosphingolipids | AD | Serum | Increased | Non-specific (MND [[54](#_ENREF_54), [55](#_ENREF_55)], C. jejuni with/without GBS [[56](#_ENREF_56)]) |
| 30 | Gangliosides (GM1, GQ1b, GT1a) [[53](#_ENREF_53), [57](#_ENREF_57), [58](#_ENREF_58)] | IgM | Sialic acid-containing glycosphingolipids | AD, VaD | Serum | With age & severity, no change | Inconsistent & Non-specific (MND [[54](#_ENREF_54), [55](#_ENREF_55)], C. jejuni with/without GBS [[56](#_ENREF_56)]) |
| 31 | Receptor for advanced glycation endproducts (RAGE) [[4](#_ENREF_4), [59](#_ENREF_59)] | IgG | Receptors | AD, MCI | Serum | Increased | Non-specific (Rheumatoid arthritis [[60](#_ENREF_60)]) |
| 32 | Oxidized low-density lipoprotein (OxLDL) [[61](#_ENREF_61)] | IgG | Lipoprotein | AD | CSF | Increased | Non-specific (SLE [[62](#_ENREF_62)], Acute myeloblastic leukaemia [[63](#_ENREF_63)], Acute coronary syndrome [[64](#_ENREF_64)]) |
| 33 | Rabaptin 5 (RABPT5) [[65](#_ENREF_65)] | IgG | GTPase binding protein | AD, SLE | Serum | Increased | Non-specific |
| 34 | Aldolase [[66](#_ENREF_66)] | IgG | Enzyme | AD | Serum | Increased | Non-specific (Atypical movement disorders [[67](#_ENREF_67)], Diabetic retinopathy [[68](#_ENREF_68)], Hepatitis [[69](#_ENREF_69)]) |

**Abbreviations:** AD: Alzheimer’s disease, VaD: Vascular dementia, SDAT: Senile dementia of Alzheimer’s type, MCI: Mild cognitive impairment, DVT: Deep vein thrombosis, RA: Rheumatoid arthritis, MND: Motor neuron disease, SLE: Systemic lupus erythematosus , GBS: Guillain-Barré syndrome, α-MSH: α-Melanocyte-stimulating hormone

**References:**

[1] Sollvander S, Ekholm-Pettersson F, Brundin RM, Westman G, Kilander L, Paulie S, et al. Increased Number of Plasma B Cells Producing Autoantibodies Against Abeta42 Protofibrils in Alzheimer's Disease. J Alzheimers Dis. 2015;48:63-72.

[2] Maftei M, Thurm F, Schnack C, Tumani H, Otto M, Elbert T, et al. Increased levels of antigen-bound beta-amyloid autoantibodies in serum and cerebrospinal fluid of Alzheimer's disease patients. PLoS One. 2013;8:e68996.

[3] Kellner A, Matschke J, Bernreuther C, Moch H, Ferrer I, Glatzel M. Autoantibodies against beta-amyloid are common in Alzheimer's disease and help control plaque burden. Annals of neurology. 2009;65:24-31.

[4] Mitchell MB, Buccafusco JJ, Schade RF, Webster SJ, Mruthinti S, Harrell DU, et al. RAGE and Abeta immunoglobulins: relation to Alzheimer's disease-related cognitive function. J Int Neuropsychol Soc. 2010;16:672-8.

[5] Gruden MA, Davidova TB, Malisauskas M, Sewell RD, Voskresenskaya NI, Wilhelm K, et al. Differential neuroimmune markers to the onset of Alzheimer's disease neurodegeneration and dementia: autoantibodies to Abeta((25-35)) oligomers, S100b and neurotransmitters. Journal of neuroimmunology. 2007;186:181-92.

[6] Moir RD, Tseitlin KA, Soscia S, Hyman BT, Irizarry MC, Tanzi RE. Autoantibodies to redox-modified oligomeric Abeta are attenuated in the plasma of Alzheimer's disease patients. J Biol Chem. 2005;280:17458-63.

[7] Wallukat G, Pruss H, Muller J, Schimke I. Functional autoantibodies in patients with different forms of dementia. PLoS One. 2018;13:e0192778.

[8] Hempel P, Heinig B, Jerosch C, Decius I, Karczewski P, Kassner U, et al. Immunoadsorption of Agonistic Autoantibodies Against alpha1-Adrenergic Receptors in Patients With Mild to Moderate Dementia. Therapeutic apheresis and dialysis : official peer-reviewed journal of the International Society for Apheresis, the Japanese Society for Apheresis, the Japanese Society for Dialysis Therapy. 2016;20:523-9.

[9] de Oliveira-Junior LC, Araujo Santos Fde A, Goulart LR, Ueira-Vieira C. Epitope Fingerprinting for Recognition of the Polyclonal Serum Autoantibodies of Alzheimer's Disease. BioMed research international. 2015;2015:267989.

[10] Giil LM, Kristoffersen EK, Vedeler CA, Aarsland D, Nordrehaug JE, Winblad B, et al. Autoantibodies Toward the Angiotensin 2 Type 1 Receptor: A Novel Autoantibody in Alzheimer's Disease. J Alzheimers Dis. 2015;47:523-9.

[11] McIntyre JA, Ramsey CJ, Gitter BD, Saykin AJ, Wagenknecht DR, Hyslop PA. Antiphospholipid autoantibodies as blood biomarkers for detection of early stage Alzheimer's disease. Autoimmunity. 2015;48:344-51.

[12] McIntyre JA, Chapman J, Shavit E, Hamilton RL, Dekosky ST. Redox-reactive autoantibodies in Alzheimer's patients' cerebrospinal fluids: preliminary studies. Autoimmunity. 2007;40:390-6.

[13] McIntyre JA, Wagenknecht DR, Ramsey CJ. Redox-reactive antiphospholipid antibody differences between serum from Alzheimer's patients and age-matched controls. Autoimmunity. 2009;42:646-52.

[14] Tuhrim S. Antiphospholipid antibodies and stroke. Current cardiology reports. 2004;6:130-4.

[15] Brey RL. Antiphospholipid antibodies and ischemic stroke. Heart disease and stroke : a journal for primary care physicians. 1992;1:379-82.

[16] Costa A, Bini P, Hamze-Sinno M, Moglia A, Franciotta D, Sinforiani E, et al. Galanin and alpha-MSH autoantibodies in cerebrospinal fluid of patients with Alzheimer's disease. Journal of neuroimmunology. 2011;240-241:114-20.

[17] Vacirca D, Delunardo F, Matarrese P, Colasanti T, Margutti P, Siracusano A, et al. Autoantibodies to the adenosine triphosphate synthase play a pathogenetic role in Alzheimer's disease. Neurobiol Aging. 2012;33:753-66.

[18] Vacirca D, Barbati C, Scazzocchio B, Masella R, Rosano G, Malorni W, et al. Anti-ATP synthase autoantibodies from patients with Alzheimer's disease reduce extracellular HDL level. J Alzheimers Dis. 2011;26:441-5.

[19] Le Naour F, Brichory F, Misek DE, Brechot C, Hanash SM, Beretta L. A distinct repertoire of autoantibodies in hepatocellular carcinoma identified by proteomic analysis. Molecular & cellular proteomics : MCP. 2002;1:197-203.

[20] Shukla S, Pranay A, D'Cruz AK, Chaturvedi P, Kane SV, Zingde SM. Immunoproteomics reveals that cancer of the tongue and the gingivobuccal complex exhibit differential autoantibody response. Cancer biomarkers : section A of Disease markers. 2009;5:127-35.

[21] Koval L, Lykhmus O, Kalashnyk O, Bachinskaya N, Kravtsova G, Soldatkina M, et al. The presence and origin of autoantibodies against alpha4 and alpha7 nicotinic acetylcholine receptors in the human blood: possible relevance to Alzheimer's pathology. J Alzheimers Dis. 2011;25:747-61.

[22] Watson R, Jepson JE, Bermudez I, Alexander S, Hart Y, McKnight K, et al. Alpha7-acetylcholine receptor antibodies in two patients with Rasmussen encephalitis. Neurology. 2005;65:1802-4.

[23] Marcello A, Wirths O, Schneider-Axmann T, Degerman-Gunnarsson M, Lannfelt L, Bayer TA. Reduced levels of IgM autoantibodies against N-truncated pyroglutamate Abeta in plasma of patients with Alzheimer's disease. Neurobiol Aging. 2011;32:1379-87.

[24] Busse S, Brix B, Kunschmann R, Bogerts B, Stoecker W, Busse M. N-methyl-d-aspartate glutamate receptor (NMDA-R) antibodies in mild cognitive impairment and dementias. Neuroscience research. 2014;85:58-64.

[25] Davydova TV, Voskresenskaya NI, Gorbatov VY, Fomina VG, Doronina OA, Maksunova IV. Production of autoantibodies to glutamate during Alzheimer's dementia. Bulletin of experimental biology and medicine. 2009;147:405-7.

[26] Davydova TV, Voskresenskaya NI, Fomina VG, Vetrile LA, Doronina OA. Induction of autoantibodies to glutamate in patients with Alzheimer's disease. Bulletin of experimental biology and medicine. 2007;143:182-3.

[27] De Bruijn MA, Titulaer MJ. Anti-NMDAR encephalitis and other glutamate and GABA receptor antibody encephalopathies. Handbook of clinical neurology. 2016;133:199-217.

[28] Trippe J, Steinke K, Orth A, Faustmann PM, Hollmann M, Haase CG. Autoantibodies to glutamate receptor antigens in multiple sclerosis and Rasmussen's encephalitis. Neuroimmunomodulation. 2014;21:189-94.

[29] Mecocci P, Parnetti L, Romano G, Scarelli A, Chionne F, Cecchetti R, et al. Serum anti-GFAP and anti-S100 autoantibodies in brain aging, Alzheimer's disease and vascular dementia. Journal of neuroimmunology. 1995;57:165-70.

[30] Jankovic BD, Djordjijevic D. Differential appearance of autoantibodies to human brain S100 protein, neuron specific enolase and myelin basic protein in psychiatric patients. The International journal of neuroscience. 1991;60:119-27.

[31] Poletaev AB, Morozov SG, Gnedenko BB, Zlunikin VM, Korzhenevskey DA. Serum anti-S100b, anti-GFAP and anti-NGF autoantibodies of IgG class in healthy persons and patients with mental and neurological disorders. Autoimmunity. 2000;32:33-8.

[32] Dale RC, Merheb V, Pillai S, Wang D, Cantrill L, Murphy TK, et al. Antibodies to surface dopamine-2 receptor in autoimmune movement and psychiatric disorders. Brain : a journal of neurology. 2012;135:3453-68.

[33] Davydova TV, Mikovskaya OI, Fomina VG, Voskresenskaya NI, Doronina OA. Induction of immune complexes and autoantibodies to serotonin and dopamine in patients with Alzheimer's disease. Bulletin of experimental biology and medicine. 2002;134:23-5.

[34] Schott K, Schaefer JE, Richartz E, Batra A, Eusterschulte B, Klein R, et al. Autoantibodies to serotonin in serum of patients with psychiatric disorders. Psychiatry research. 2003;121:51-7.

[35] Terryberry JW, Thor G, Peter JB. Autoantibodies in neurodegenerative diseases: antigen-specific frequencies and intrathecal analysis. Neurobiol Aging. 1998;19:205-16.

[36] Tanaka J, Nakamura K, Takeda M, Tada K, Suzuki H, Morita H, et al. Enzyme-linked immunosorbent assay for human autoantibody to glial fibrillary acidic protein: higher titer of the antibody is detected in serum of patients with Alzheimer's disease. Acta Neurol Scand. 1989;80:554-60.

[37] P ang Z, Kushiyama A, Sun J, Kikuchi T, Yamazaki H, Iwamoto Y, et al. Glial fibrillary acidic protein (GFAP) is a novel biomarker for the prediction of autoimmune diabetes. FASEB journal : official publication of the Federation of American Societies for Experimental Biology. 2017;31:4053-63.

[38] Cheng Y, Zhao X, Chen Y, Li Y, Jia R, Zhu L, et al. Circulating immune complexome analysis identified anti-tubulin-alpha-1c as an inflammation associated autoantibody with promising diagnostic value for Behcet's Disease. PLoS One. 2018;13:e0199047.

[39] Zhao X, Cheng Y, Gan Y, Jia R, Zhu L, Sun X. Anti-tubulin-alpha-1C autoantibody in systemic lupus erythematosus: a novel indicator of disease activity and vasculitis manifestations. Clinical rheumatology. 2018;37:1229-37.

[40] Svarcova J, Fialova L, Bartos A, Steinbachova M, Malbohan I. Cerebrospinal fluid antibodies to tubulin are elevated in the patients with multiple sclerosis. European journal of neurology. 2008;15:1173-9.

[41] Mecocci P, Ekman R, Parnetti L, Senin U. Antihistone and anti-dsDNA autoantibodies in Alzheimer's disease and vascular dementia. Biol Psychiatry. 1993;34:380-5.

[42] Sun XY, Shi J, Han L, Su Y, Li ZG. Anti-histones antibodies in systemic lupus erythematosus: prevalence and frequency in neuropsychiatric lupus. Journal of clinical laboratory analysis. 2008;22:271-7.

[43] Maruyama I, Ikeda Y, Nakazawa M, Ohguro H. Clinical roles of serum autoantibody against neuron-specific enolase in glaucoma patients. The Tohoku journal of experimental medicine. 2002;197:125-32.

[44] Philpot M, Colgan J, Levy R, Holland A, Mirakian R, Richardson CA, et al. Prolactin cell autoantibodies and Alzheimer's disease. J Neurol Neurosurg Psychiatry. 1985;48:287-8.

[45] van Dorp TA, Endtz LJ, te Velde J, Slaets JP, Reichgelt J. Prolactin cell autoantibodies and Alzheimer's disease. J Neurol Neurosurg Psychiatry. 1985;48:1308-9.

[46] Jara LJ, Medina G, Saavedra MA, Vera-Lastra O, Torres-Aguilar H, Navarro C, et al. Prolactin has a pathogenic role in systemic lupus erythematosus. Immunologic research. 2017;65:512-23.

[47] Fojtikova M, Tomasova Studynkova J, Filkova M, Lacinova Z, Gatterova J, Pavelka K, et al. Elevated prolactin levels in patients with rheumatoid arthritis: association with disease activity and structural damage. Clinical and experimental rheumatology. 2010;28:849-54.

[48] Bartos A, Fialova L, Svarcova J. Lower Serum Antibodies Against Tau Protein and Heavy Neurofilament in Alzheimer's Disease. J Alzheimers Dis. 2018;64:751-60.

[49] Rosenmann H, Meiner Z, Geylis V, Abramsky O, Steinitz M. Detection of circulating antibodies against tau protein in its unphosphorylated and in its neurofibrillary tangles-related phosphorylated state in Alzheimer's disease and healthy subjects. Neuroscience letters. 2006;410:90-3.

[50] Bartos A, Fialova L, Svarcova J, Ripova D. Patients with Alzheimer disease have elevated intrathecal synthesis of antibodies against tau protein and heavy neurofilament. Journal of neuroimmunology. 2012;252:100-5.

[51] Lin CY, Sheu JJ, Tsai IS, Wang ST, Yang LY, Hsu IU, et al. Elevated IgM against Nepsilon-(Carboxyethyl)lysine-modified Apolipoprotein A1 peptide 141-147 in Taiwanese with Alzheimer's disease. Clinical biochemistry. 2018;56:75-82.

[52] Giil LM, Vedeler CA, Kristoffersen EK, Nordrehaug JE, Heidecke H, Dechend R, et al. Antibodies to Signaling Molecules and Receptors in Alzheimer's Disease are Associated with Psychomotor Slowing, Depression, and Poor Visuospatial Function. J Alzheimers Dis. 2017;59:929-39.

[53] Hatzifilippou E, Koutsouraki E, Costa VG, Baloyannis SJ. Antibodies against gangliosides in patients with dementia. Am J Alzheimers Dis Other Demen. 2014;29:660-6.

[54] Shy ME, Heiman-Patterson T, Parry GJ, Tahmoush A, Evans VA, Schick PK. Lower motor neuron disease in a patient with autoantibodies against Gal(beta 1-3)GalNAc in gangliosides GM1 and GD1b: improvement following immunotherapy. Neurology. 1990;40:842-4.

[55] Jauberteau MO, Gualde N, Preud'Homme JL, Rigaud M, Gil R, Vallat JM, et al. Human monoclonal IgM with autoantibody activity against two gangliosides (GM1 and GD1b) in a patient with motor neuron syndrome. Clinical and experimental immunology. 1990;80:186-91.

[56] von Wulffen H, Hartard C, Scharein E. Seroreactivity to Campylobacter jejuni and gangliosides in patients with Guillain-Barre syndrome. The Journal of infectious diseases. 1994;170:828-33.

[57] Miura Y, Miyaji K, Chai YL, Chen CL, Lai MK, Yuki N. Autoantibodies to GM1 and GQ1balpha are not biological markers of Alzheimer's disease. J Alzheimers Dis. 2014;42:1165-9.

[58] Hatzifilippou E, Koutsouraki E, Banaki T, Traka M, Costa VG, Baloyannis SJ. Antibodies against GM1 in demented patients. Am J Alzheimers Dis Other Demen. 2008;23:274-9.

[59] Mruthinti S, Schade RF, Harrell DU, Gulati NK, Swamy-Mruthinti S, Lee GP, et al. Autoimmunity in Alzheimer's disease as evidenced by plasma immunoreactivity against RAGE and Abeta42: complication of diabetes. Curr Alzheimer Res. 2006;3:229-35.

[60] Chen YS, Yan W, Geczy CL, Brown MA, Thomas R. Serum levels of soluble receptor for advanced glycation end products and of S100 proteins are associated with inflammatory, autoantibody, and classical risk markers of joint and vascular damage in rheumatoid arthritis. Arthritis research & therapy. 2009;11:R39.

[61] Kankaanpaa J, Turunen SP, Moilanen V, Horkko S, Remes AM. Cerebrospinal fluid antibodies to oxidized LDL are increased in Alzheimer's disease. Neurobiology of disease. 2009;33:467-72.

[62] Bassi N, Zampieri S, Ghirardello A, Tonon M, Zen M, Beggio S, et al. oxLDL/beta2GPI complex and anti-oxLDL/beta2GPI in SLE: prevalence and correlates. Autoimmunity. 2009;42:289-91.

[63] Li H, Diao YT, Li HQ, Ma Q, Cui J, Zhou YZ, et al. The association between serum levels of oxLDL-lgG and oxLDL-lgM autoantibody with adult acute myeloblastic leukaemia. Lipids in health and disease. 2010;9:11.

[64] Medeiros AM, von Muhlen CA, Gidlund MA, Bodanese R, Gottlieb MG, Bodanese LC. Antibodies against oxLDL and acute coronary syndrome. Arquivos brasileiros de cardiologia. 2010;95:47-54.

[65] Delunardo F, Margutti P, Pontecorvo S, Colasanti T, Conti F, Rigano R, et al. Screening of a microvascular endothelial cDNA library identifies rabaptin 5 as a novel autoantigen in Alzheimer's disease. Journal of neuroimmunology. 2007;192:105-12.

[66] Mor F, Izak M, Cohen IR. Identification of aldolase as a target antigen in Alzheimer's disease. J Immunol. 2005;175:3439-45.

[67] Privitera D, Corti V, Alessio M, Volonte MA, Lampasona V, Comi G, et al. Proteomic identification of aldolase A as an autoantibody target in patients with atypical movement disorders. Neurological sciences : official journal of the Italian Neurological Society and of the Italian Society of Clinical Neurophysiology. 2013;34:313-20.

[68] Ahn BY, Song ES, Cho YJ, Kwon OW, Kim JK, Lee NG. Identification of an anti-aldolase autoantibody as a diagnostic marker for diabetic retinopathy by immunoproteomic analysis. Proteomics. 2006;6:1200-9.

[69] Brown C, Toh BH, Pedersen JS, Clarke FM, Mackay IR, Gust I. Autoantibody to aldolase in acute and chronic hepatitis. Pathology. 1987;19:347-50.
